# Dental Pulp-Derived Stem Cells Reduce Inflammation, Accelerate Wound Healing and Mediate M2 Polarization of Myeloid Cells

**DOI:** 10.3390/biomedicines10081999

**Published:** 2022-08-17

**Authors:** Sarah Anderson, Prateeksha Prateeksha, Hiranmoy Das

**Affiliations:** Department of Pharmaceutical Sciences, Jerry H. Hodge School of Pharmacy, Texas Tech University Health Sciences Center, Amarillo, TX 79106, USA

**Keywords:** dental pulp-derived stem cells, monocytes, M2, polarization, inflammation, priming

## Abstract

This work aimed to validate the potential use of dental pulp-derived stem cells (DPSCs) for the treatment of inflammation by defining their mechanisms of action. We planned to investigate whether priming of DPSC with proinflammatory molecules had any impact on their behavior and function. In the first step of our validation in vitro, we showed that priming of DPSCs with the bioactive agents LPS, TNF-α, or IFN-γ altered DPSCs’ immunologic properties by increasing their expression levels of IL-10, HGF, IDO, and IL-4 and by decreasing their mitochondrial functions. Moreover, DPSCs induced accelerated wound healing irrespective of priming, as determined by using a gut epithelial cell line in a scratch wound assay. Wound healing of gut epithelial cells was mediated by regulating the expressions of AKT, NF-κB, and ERK1/2 proteins compared to the control epithelial cells. In addition, primed DPSCs altered monocyte polarization toward an immuno-suppressive phenotype (M2), where monocytes expressed higher levels of IL-4R, IL-6, Arg1, and YM-1 compared to monocytes cultured with control DPSCs. In silico analysis revealed that this was accomplished in part by the interaction between kynurenine and PPARγ, which regulated the expression of M2 differentiation-related genes. Collectively, these data provided evidence that the DPSCs reduced inflammation, induced M2 polarization of myeloid cells, and healed damaged gut epithelial cells through inactivation of inflammation and modulating constitutively active signaling pathways.

## 1. Introduction

Stem cells have been studied for decades. These studies have focused on understanding embryonic growth, growing tissues and organs, and developing therapeutic options for different diseases [1,2,3]. However, the FDA, currently, has only approved hematopoietic stem cells from umbilical cord blood as a therapeutic product. Hematopoietic stem cells from bone marrow are also in current use, but are not regulated by the FDA. These stem cells are used in disorders affecting the hematopoietic system that are inherited, acquired, or result from myeloablative treatment. Examples of such disorders include acute myelogenous leukemia, anemia, sickle cell disease, or complications from chemotherapy [4]. However, there are many other diseases and types of stem cells. Stem cells can be broadly separated into three categories: embryonic, adult, and induced pluripotent. Embryonic stem cells can differentiate into any kind of cell and are sourced from embryos. They are not used in therapy due to the formation of teratoma-like tumors that have occurred after transplantation [5]. Induced pluripotent cells (iPSC) are mostly fully-differentiated cells that have been forced to express transcription factors, initially Oct4, Sox2, Klf4, and c-Myc and, currently, various stem cell factors. This exposure causes the cell to dedifferentiate and become pluripotent. iPSCs have been studied for cell transplantation therapy, disease modeling, and drug screening [5,6]. Adult stem cells include bone marrow-derived mononuclear cells, hematopoietic stem cells, mesenchymal stem cells, resident adult stem cells, and umbilical cord-derived stem cells [7]. Adult stem cells are placed into these categories because they are partially differentiated. They can only become a small number of different cell types, compared to embryonic cells, which can become any cell type [8]. 

Literature has shown that priming mesenchymal stem cells (MSCs) makes them more efficacious in their immunomodulatory functions. These studies were conducted in various autoimmune disease models in mice for graft versus host disease and atopic dermatitis [9,10,11]. During inflammation, immune cells and damaged cells release pro-inflammatory cytokines, leading to a severely inflamed microenvironment. Additionally, if the area is exposed to the outside environment, proinflammatory antigens, such as LPS, come into play, as seen in inflammatory bowel disease or chronic ulcers [12]. By priming the stem cells, cells change the activity of different molecular pathways so that they can be more efficacious in this proinflammatory microenvironment. The pathway that is activated results in very distinct responses in MSCs. For example, it was shown that, depending on which toll-like receptor (TLR) were activated, MSCs had very different immunomodulatory responses. When TLR3 was activated, T-cell activation was suppressed; the MSCs had higher expression of cytokines such as IL-4, IP-10, RANTES, and IL-1Rα. These MSCs also provided greater deposition of fibronectin and increased expression of IDO and PGE2. Alternatively, TLR4-primed MSCs had higher expression of cytokines such as IL-6 and IL-8, deposited greater amounts of collagen I/II, and did not suppress T-cell activation [13]. Furthermore, it was shown that one set of MSCs primed with IFN-γ and another set of MSCs primed with polyinosinic-polycytidylic acid (poly(I:C)) showed similar therapeutic effects in the animal model. However, bioinformatic analyses showed distinct differences in what factors were expressed depending on what the MSCs were primed with. MSCs primed with poly(I:C) had greater activation of pathways associated with apoptosis of phagocytes, myeloid cells, and leukocytes, as well as cytotoxicity of cells. MSCs primed with IFN-γ showed changes in pathways associated with cell death and survival, cellular development, cell growth and proliferation, cellular movement, and cell-to-cell signaling and interactions [10].

Immune cells such as T-cells, B-cells, macrophages, and neutrophils play a critically important role in the pathogenesis of many diseases. The polarization of macrophages is determined by the local microenvironment. Macrophages can be polarized toward a proinflammatory M1 phenotype or an anti-inflammatory M2 phenotype. M1 polarization is induced by cytokines such as IFN-γ and TNF-α and produces cytokines such as IL-1β, TNF-α, and IL-23, whereas M2 polarization is influenced by cytokines such as IL-4 and IL-13 and produces proteins such as arginase-1, IL-10, and TNF-β [14]. M1 polarization is important in the early stages of infection and disease to help protect the body. However, after M1 polarization has completed its task, M2 polarization is needed to facilitate wound healing and stop the inflammatory response [15]. It has been shown that priming MSCs can induce the M2 polarization using a retinoic acid as a priming agent [16]. This further supports the use of priming as a tool to enhance the function of stem cells.

Many studies evaluating the potential of MSCs as a therapy have used stem cell sources such as umbilical cords, bone marrow, or adipose tissue [17]. In our current study, we used dental pulp-derived stem cells (DPSCs), which are extracted from third molars and share many characteristics with other MSCs. Using DPSCs instead of other MSC sources has several advantages. First, wisdom teeth are considered routine medical waste, so, unlike stem cells taken from umbilical cord blood or bone marrow, there is no limit of fresh stem cells due to lack of donations. Second, wisdom tooth extraction is a significantly cheaper and more common surgery than liposuction, by which adipose-derived MSCs are obtained. We hypothesized that priming DPSCs with proinflammatory factors would make them more efficacious for the treatment of inflammation. In this study, we tested this hypothesis by evaluating the DPSCs’ anti-inflammatory profile, their ability to induce M2 polarization in macrophages, and their ability to promote wound closure in gut epithelial cells.

## 2. Materials and Methods

### 2.1. Materials

RAW264.7 and LoVo cells were purchased from ATCC. Cells were cultured in DMEM supplemented with 10% FBS and 1% antibiotic and antimycotic solutions. LPS, TNF-α, and IFN-γ were purchased from Peprotech, Cranbury, NJ, USA. Kynurenine was purchased from Sigma Aldrich, St. Louis, MO, USA. 

### 2.2. Isolation and Culture of DSPCs

Isolation and culture of DPSCs were carried out as previously described [18]. Briefly, third molars were obtained from local dental clinics and rinsed three times with phosphate-buffered saline (PBS), with 1% penicillin/streptomycin/glutamine (PSG) or 1% anti-biotic, and anti-mycotic solutions (all from Gibco, Thermo Fisher, Waltham, MA, USA). Dental pulp was extracted, minced into 1 mm cubes, and plated on 60 mm culture dishes with α-Modified Eagle Medium (MEM) (Gibco) containing 20% Fetal Bovine Serum (Hyclone, Thermo Fisher, Waltham, MA, USA) and 1% PSG. Cells migrating from the pulp were detached and plated as passage 1 cells. Homogenous cell populations were confirmed via flow cytometry. Cells from passage 3 to passage 9 were used for experiments.

### 2.3. Multiplex ELISA

DPSCs were cultured in α-MEM with 20% FBS in a 24-well plate at a density of 5 × 10^5^ cells/well for 24 h. Collected supernatants were mailed to Ray Biotech and the medium without any DPSCs was used as a control. Each of the samples was in duplicate, and analysis was performed four times. Statistical analysis was done with the raw data and the standard curves provided.

### 2.4. Priming of DPSCs

DPSCs were primed with either 10 ng/mL LPS, 10 ng/mL TNF-α, or 100 ng/mL IFN-γ for 4 h or 24 h in α-MEM containing 20% FBS and 1% PSG in a 37 °C incubator with 5% CO_2_. The control group consisted of unprimed DPSCs in α-MEM containing 20% FBS and 1% PSG. Isolated cells were harvested for quantitative RT-PCR and Western blot analysis. All experiments were performed in triplicate.

### 2.5. Flow Cytometry

The fluorescently-labeled antibodies for cell surface markers were CD73, CD90, and CD105. They were purchased from the established vendor (eBioscience, San Diego, CA, USA). Flow cytometry was carried out by washing the DPSCs three times with 1× PBS. Cells were then incubated at 4 °C for more than 30 min in presence of one of the antibodies. After antibody staining, cells were washed three times with 1 × PBS. Flow cytometric analysis was performed using a FACSVerse flow cytometer (BD Biosciences, East Rutherford, NJ, USA). At least 10,000 events were acquired for each sample for analysis using BD FACSuite software (BD Biosciences, East Rutherford, NJ, USA).

### 2.6. DPSC and RAW264.7 Co-Culture

Before co-culture, DPSCs were primed with either LPS, TNF-α, or IFN-γ for four hours. DPSCs and RAW264.7 cells were co-cultured in a trans-well system keeping DPSC within 22 μm apical inserts and RAW264.7 in the bottom of the well of a 6-well culture plate with a 1:10 ratio of DPSCs to RAW264.7 cells. The control groups were not co-cultured with DPSCs. The time points were selected as 12 h and 24 h for harvesting. After each time point, protein and mRNA were collected from the cultured RAW264.7 cells. The protein was used for Western blots. The mRNA was used for qRT-PCR analysis. All experiments were performed in triplicate.

### 2.7. Real-Time RT-PCR Analysis

Total RNA was isolated from DPSCs, DPSCs primed with LPS, DPSCs primed with TNF-α, DPSCs primed with IFN-γ, monocytes, and monocytes co-cultured with DPSCs and primed DPSCs. One microgram of RNA was used for the synthesis of cDNA using an oligo dT (Invitrogen, Thermo-Fisher) primer. RT-PCR was performed using one μL of cDNA with gene-specific primers for HGF, IL-4, IDO, IL-10, and GAPDH (human), as well as IL-1β, TNF-α, Arg-1, YM-1, IL-6, and IL-4R, and β-actin (murine) (Supplementary Tables S1 and S2). A standard SYBR green Taqman protocol and real-time PCR machine (Stratagene, MX3000P, Santa Clara, CA, USA) were used. The relative expression levels of the chosen genes were measured against respective unstimulated cells. Experiments were performed in triplicate.

### 2.8. Western Blotting

To perform Western blot (WB), the cells were lysed in 60 μL precooled RIPA lysis buffer (Millipore Sigma Aldrich Corporation, 20-188) for 30 min on ice and centrifuged at 13,200× *g* for 15 min. The supernatant was collected, and protein concentration was estimated with Bradford’s reagent (Bio-Rad Incorporation, Berkley, CA, USA, 5000006) using bovine serum albumin (BSA) (Sigma Aldrich Corporation, St. Louis, MO, USA, A7906-10G) as a standard. Equal amounts of protein (40 μg) were separated by 10–12% SDS-PAGE gel electrophoresis and transferred to a polyvinylidene difluoride membrane (Bio-Rad Incorporation, Berkley, CA, USA, 1620115). After blocking with 5% milk for 30 min at room temperature, the membranes were incubated with primary antibodies for 12–16 h at 4 °C. Then, membranes were re-incubated with appropriate HRP (horseradish peroxidase)-labeled secondary antibodies (Cell Signaling Technology, Danvers, MA, USA 7074, and 7076) for 1–2 h at room temperature. Immunoreactive protein bands were visualized by enhanced chemiluminescence (ECL, Amersham Pharmacia Biotechnology, Uppsala, Sweden, RPN2232), and band detections were performed within the linear range. Total protein analysis was performed with equal amounts of proteins isolated from RAW264.7 cells, LoVo cells, and DPSCs. Western blot analysis was performed using the following antibodies: pp65 (cat # 3033, Santa Cruz, CA, USA), p65 (cat# 8242, Santa Cruz, CA, USA), pAKT (cat# 3787, Cell Signaling, Danvers, MA, USA), AKT (cat# 4695, Cell Signaling, Danvers, MA, USA), pERK1/2 (cat# 4370, Cell Signaling, Danvers, MA, USA), ERK1/2 (cat# 4695, Cell Signaling, Danvers, MA, USA), Nos-2 (cat # 2977, Cell Signaling, Danvers, MA, USA), IDO (cat# 86630, Cell Signaling, Danvers, MA, USA), YM-1 (cat# ab93034, Abcam, Cambridge, UK), Arg-1 (cat# gtx109242, Genetex, Irvine, CA, USA), and GAPDH as a loading control (cat# 2118, Cell Signaling, Danvers, MA, USA). 

### 2.9. Immunocytochemistry

RAW264.7 cells were seeded on coverslips in a six-well plate at 75,000 cells/well. After allowing the cells to adhere overnight, RAW264.7 cells were co-cultured with DPSCs, DPSCs either primed with LPS, TNF-α, IFN-γ, or 20 μM kynurenine. After 24 h RAW264.7 cells were washed with 1× PBS three times. Next, the cells were fixed with 4% paraformaldehyde in PBS (Chem Cruz, Santa Cruz Biotechnology, Santa Cruz, CA, USA) and permeabilized with 0.2% Triton X-100. The cells were stained with the primary antibodies LAT-1 (cat# 374232, Santa Cruz Biotechnology, Santa Cruz, CA, USA) and PPARγ (cat# 2435, Cell Signaling, Danvers, MA, USA) and AlexaFluor conjugated secondary antibodies were used (cat# A11001, and A21244, Invitrogen Corporation, Carlsbad, CA, USA). Immunofluorescence was observed using a confocal microscope (Nikon A1MP Confocal/Multiphoton Microscope using NIS Elements v4.30.01, Tokyo, Japan) on the FITC, TRITC, and DAPI channels.

### 2.10. Scratch Wound Assay

The human gut cell line, LoVo, was used for a scratch wound-healing assay. Before co-culture with the gut epithelial cells, DPSCs were primed with either LPS, TNF-α, or IFN-γ for four hours in DMEM containing 1% FBS. At the end of the time point, the media and stimulants were completely removed by thoroughly washing three times with 1× PBS, and fresh media (DMEM containing 1% FBS) was added. Directly before co-culture with DPSC (with or without priming), the epithelial cells were scratched with a 200 µL pipette tip. LoVo cells were co-cultured in a trans-well system with a 1:10 ratio of DPSCs, either primed or without priming, to LoVo cells in a 6-well cell culture plate. Control LoVo cells were not co-cultured with DPSCs. The control gut epithelial cell culture had no DPSC treatment. The cells were kept in a 37 °C incubator with 5% CO_2_ until full wound closure was observed. Live-cell images were taken using a bright-field microscope once a day until one of the groups showed wound closure (6–7 days). At the end of the experiment, the protein was isolated from the gut epithelial cells for WB. Cell proliferation was measured using the MRI wound-healing macro for ImageJ. MRI wound healing tool measured the area of the wound. The formula below was used to measure wound closure.

$$\frac{Area\;at\;0h-Area}{Area\;at\;0h}\;\times \;100\%$$

Error bars were calculated using standard error of the mean and Student’s *t*-test was used to calculate significance. The value *p* ˂ 0.05 was considered significant. 

### 2.11. Mitostress Analysis

DPSCs were seeded at a density of 2 × 10^5^ cells per well. Cells were counted and plated in triplicate in the Seahorse cell culture plate and allowed to adhere for 24 h. Control DPSCs were not stimulated. Primed DPSCs were stimulated with either LPS (10 ng/mL), TNF-α (10 ng/mL), or IFN-γ (100 ng/mL) for 4 h and then were rinsed with 1× PBS three times. Fresh medium was then added and cultured for 24 h, after which the cells were prepared for the Seahorse MitoStress test following the Agilent Seahorse SF Cell Mito Stress Test Kit User Guide. 

Briefly, assay media were prepared using Seahorse XF DMEM (Agilent, Santa Clara, CA, USA), supplemented with 1 mM pyruvate (Agilent, Santa Clara, CA, USA), 2 mM glutamine (Agilent, Santa Clara, CA, USA), and 10 mM glucose (Agilent, Santa Clara, CA, USA). Then, oligomycin, 2-[2-[4-(trifluoromethoxy) phenyl] hydrazinylidene]-propanedinitrile (FCCP), and rotenone/antimycin A were diluted in media making the following concentrations: 1 µM oligomycin, 1 µM FCCP, and 0.5 µM rotenone/antimycin A. On the day of the assay, DPSC culture media were removed, and each well was rinsed with assay media at least 3 times. Next, 500 µL of assay medium was added to each well. Cells were incubated in a non-CO_2_ incubator at 37 °C for one hour before running the assay. The assay was run using a standard, unmodified MitoStress Test protocol from Agilent in an Agilent XFe24 machine.

### 2.12. Kynurenine Assay

DPSCs were plated and allowed to adhere overnight. The next day, they were primed for 4 h with either LPS, TNF-α, or IFN-γ. The cells were washed 3 × with PBS and a fresh medium was added. Following the protocol from Richards et al., 140 µL of media was removed from each sample, placed into a 96 well plate, and incubated with 10 µL of 6.1 N trichloroacetic acid at 50 °C for 30 min. The plate was then centrifuged for 10 min at 2500 rpm. 100 µL of supernatant was transferred to a different well and mixed with 100 µL of freshly prepared Ehrlich’s reagent. After 10 min of incubation at room temperature, absorbance was read at 490 nm using a microplate reader [19].

### 2.13. In Silico Modeling

In silico analysis was performed with AutoDock 4.2 software to understand the interaction of IDO and its downstream molecules like kynurenine and PPARγ. The X-ray crystallographic structure of PPARγ (PDB ID: 2ZNO) was downloaded from RCSB Protein Data Bank (https://www.rcsb.org/ accessed on 5 February 2022). The chemical structures of Kynurenine and Rosiglitazone, a PPARγ agonist, were retrieved from PubChem (https://pubchem.ncbi.nlm.nih.gov/ accessed on 5 February 2022). The protein and ligands were primed with UCSF Chimera software and open Babel, an online tool in the (.pdb) file format. Next, the Kollman Charge was added to the protein structure and saved in the (.pdbqt) file. The ligand-binding pocket of PPARγ was defined by sketching a 3D grid box with the dimensions, 126, 126, and 98 Å. The x, y, and z centers for PPARγ were also determined. The docking file (.dpf) was prepared by implementing the Lamarckian genetic algorithm (GA 4.2) with all default settings. The prepared file (.dpf) was executed through AutoDock 4.2, (Scripps Research, San Diego, CA, USA) scripts resulting in the (.dlg) file. All poses of the docked protein-ligand complex were evaluated based on binding energy (ΔGbind) expressed as kcal/mol. The in-depth interactions of kynurenine and rosiglitazone with PPARγ were visualized with Biovia Discovery Studio Visualizer 4.5 (San Leandro, CA, USA).

### 2.14. Statistical Analysis

These results are expressed as mean ± SEM. Student’s *t*-test was used to calculate statistical significance. Statistical significance was defined as * = *p* ˂ 0.05. Columns and error bars in the figures show the results of experiments run in triplicate.

## 3. Results

### 3.1. DPSCs Secrete Anti-Inflammatory Proteins

**Figure 1:**
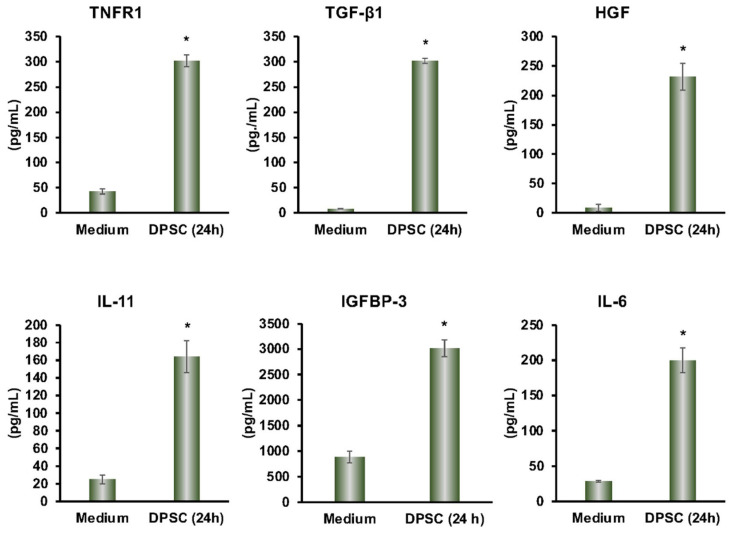
DPSCs secrete the anti-inflammatory proteins TNFR1, TGF-β1, HGF, IL-11, IGFBP-3, and IL-6 after 24 h in media as measured by Multiplex ELISA. Culture media by itself is the control. Statistical significance was defined as (*) = *p* ˂ 0.05.

From the multiplex ELISA (RayBiotech Life, Atlanta, GA, USA) analysis, we found a variety of proteins that were secreted by DPSCs in cultured media. The secreted proteins TGF-β, IL-11, TNF-R1, HGF, IGFBP-3, and IL-6 had anti-inflammatory effects on a variety of cell types. This showed that our DPSCs had the same anti-inflammatory activity as other MSCs (Figure 1).

### 3.2. Effect of Priming of MSC Markers in DPSCs

Our previous findings showed that DPSCs were positive for the MSC markers CD90, CD105, and CD73 [18]. We found that priming with LPS, TNF-α, or IFN-γ had no significant effect on the expression of these MSC markers (Supplementary Figure S1).

### 3.3. Effect of Priming on Mitochondrial Health in DPSCs

**Figure 2:**
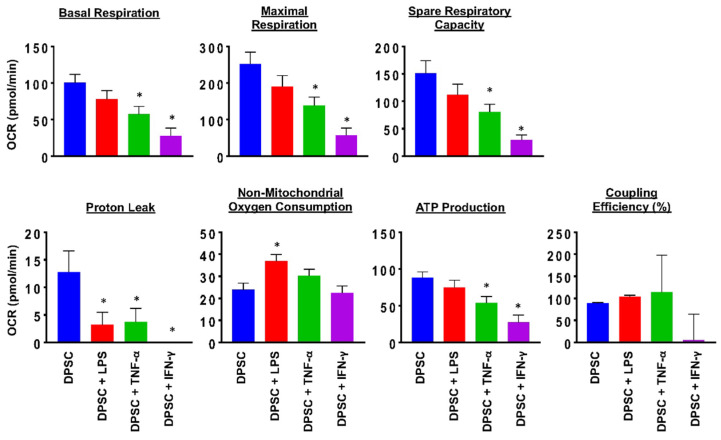
DPSCs stimulated with TNF-a (10 ng/mL) and DPSCs stimulated with IFN-y (100 ng/mL) show decreased basal respiration, maximal respiration, spare respiratory capacity, proton leak, and ATP production compared to unprimed DPSCs. DPSCs stimulated with LPS (10 ng/mL) show increased nonmitochondrial oxygen consumption compared to unprimed DPSCs. Statistical significance was defined as (*) = *p* ˂ 0.05.

To evaluate the health of the DPSCs, we performed a MitoStress test using a flux analyzer. This test measured the robustness and health of the cell’s mitochondria. The results showed that 24 h after priming DPSCs for 4 h with various stimulants, there was a significant decline in mitochondrial function of DPSCs stimulated with TNF-α or IFN-γ. This was reflected in decreased basal respiration, maximal respiration, ATP production, proton leak, and spare respiratory capacity. DPSCs stimulated with LPS also showed decreased proton leak and increased nonmitochondrial oxygen consumption (Figure 2 and Figure S2). These changes suggested that priming the DPSCs negatively impacted the health of the cell by decreasing its ability to produce ATP and respond to metabolic demands. 

### 3.4. Effect of Priming on Anti-inflammatory Genes in DPSCs

**Figure 3:**
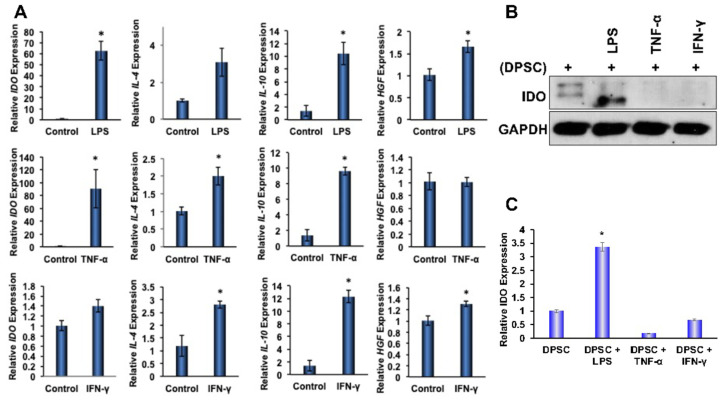
(**A**) DPSCs stimulated with LPS (10 ng/mL) showed a 60-fold increased gene expression of IDO, a 10-fold increased gene expression of IL-10, and a 1.5-fold increased expression of HGF. DPSCs stimulated with TNF-a (10 ng/mL) showed a 2-fold increased gene expression of IL-4, a 10-fold increased expression of IL-10, and a 90-fold increased expression of IDO. DPSCs stimulated with IFN-y (100 ng/mL) showed a 3-fold increased gene expression of IL-4, a 12-fold increased expression of IL-10, and a 1.4-fold increased expression of HGF. (**B**,**C**) Western Blot and densitometric analysis of the western blot show that DPSCs stimulated with LPS showed a 3-fold increase in expression of IDO. Statistical significance was defined as (*) = *p* ˂ 0.05.

To determine the effects of priming on anti-inflammatory gene expressions in DPSCs, stem cells were primed for 4 and 24 h with LPS, TNF-α, or IFN-γ, which were found to be enriched in the microenvironment of IBD. Quantitative RT-PCR analysis showed significantly increased gene expression of anti-inflammatory proteins IL-4, IL-10, HGF, and IDO in the DPSCs that were primed for 4 h or 24 h. Priming with LPS for 4 h also showed statistically significant (*p* ˂ 0.05) increases in the gene expression of IDO, IL-10, and HGF. Similarly, priming with TNF-α for 4 h also showed significantly increased gene expression of IDO, IL-4, and IL-10. In addition, priming with IFN-γ also showed significant increases in the gene expression of IL-4, IL-10, and HGF. These data confirmed that the priming of DPSCs with any of the above inflammatory factors for 4 h enhanced anti-inflammatory gene expressions in DPSCs when compared to unprimed DPSCs without maintaining a particular profile (Figure 3A). After 24 h priming, some of the anti-inflammatory gene expressions were reduced (data not shown). Therefore, in our subsequent experimental designs, we used 4 h of priming and continued with all the inflammatory factors tested, as the results were consistent. 

### 3.5. Effect of Primed DPSCs on Wound Healing of Gut Epithelial Cells

**Figure 4:**
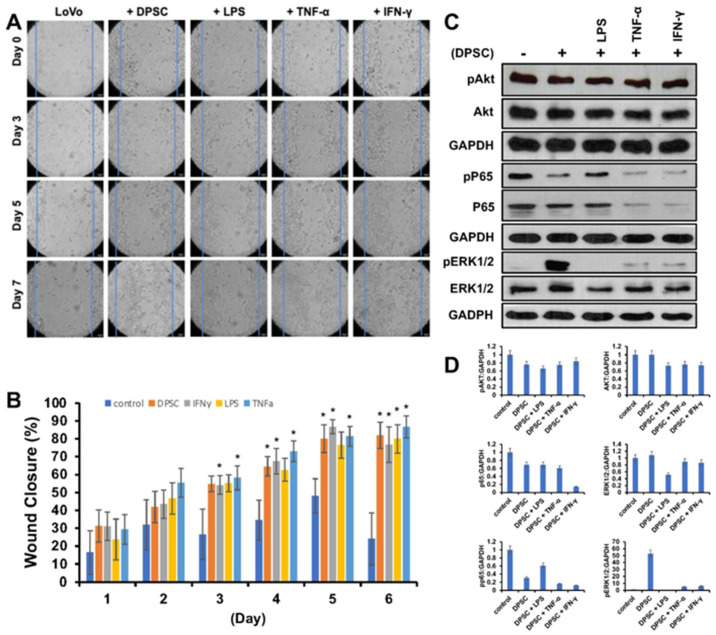
(**A**) LoVo cells co-cultured with DPSCs and primed DPSCs showed significantly greater wound closure compared to the control. (**B**) Starting on day 3, LoVo cells co-cultured with DPSCs primed with IFN-y and DPSCs primed with TNF-a were significantly more closed compared to control. On day 4, LoVo cells co-cultured with DPSCs were significantly more closed compared to control. On day 5, LoVo cells co-cultured with DPSCs primed with LPS were significantly more closed compared to control. These significant increases continued until the last time point on day 7. Statistical significance was defined as (*) = *p* < 0.05. (**C**,**D**) Western blot and densitometric analysis of the Western blot showed decreased expression of pAkt in cells co-cultured with DPSCs by 0.3-fold and cells cocultured with DPSCs primed with LPS by 0.4-fold, in cells co-cultured with DPSCs primed with TNF-α 0.3-fold, and in cells co-cultured with DPSCs primed with IFN-γ 0.2-fold. Akt levels decreased in cells co-cultured with primed DPSCs by 0.3-fold. Pp65 was decreased in cells cocultured with DPSCs by 0.6-fold and decreased in cells co-cultured with DPSCs primed with LPS by 0.4-fold, in cells co-cultured with DPSCs primed with TNF-α 0.8-fold, and in cells co-cultured with DPSCs primed with IFN-γ 0.8-fold. PERK 1/2 was increased in cells cocultured with DPSCs by 50-fold, DPSCs primed with TNF-α by 5-fold, and DPSCs primed with IFN-γ by 6-fold. ERK 1/2 was decreased in cells co-cultured with DPSCs primed with LPS by 0.5-fold.

Next, we wanted to evaluate the effects of primed DPSCs on proliferation and wound healing of LoVo gut epithelial cells. We co-cultured them with DPSCs that were either primed with LPS, TNF-α or IFN-γ for 4 h or unprimed. The epithelial cells showed significantly greater cell proliferation and greater wound healing when co-cultured with DPSCs or primed DPSCs when compared to epithelial cells alone without co-culture with any stem cells. Starting on day 3, epithelial cells co-cultured with DPSCs primed with TNF-α and epithelial cells co-cultured with DPSCs primed with IFN- γ showed 20% greater wound closure compared to control. On day 4, epithelial cells co-cultured with DPSCs showed significantly increased wound closure with 30% greater wound closure compared to control. Epithelial cells that were co-cultured with DPSCs primed with TNF-α or IFN-γ continued to show significantly increased wound closure by at least 20% until the end of the experiment. Epithelial cells co-cultured with DPSCs primed with LPS showed significantly greater wound closure starting on day 5 with 40% greater wound closure when compared to the control (Figure 4A,B).

The WB analysis revealed that levels of pAKT and pp65 decreased when epithelial cells were co-cultured with DPSCs either primed or unprimed. The levels of pERK 1/2 were increased in epithelial cells co-cultured with DPSCs and DPSCs primed with TNF-α or IFN-γ (Figure 4C,D). 

### 3.6. Effect of Primed DPSCs on Monocyte Polarization-Related Gene Expression

**Figure 5:**
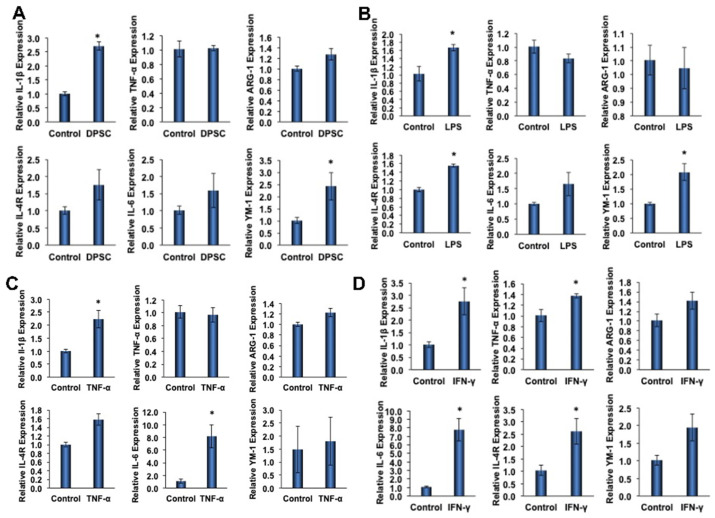
(**A**) RAW264.7 cells co-cultured with DPSCs showed 3-fold increased gene expression of IL-1B. There was increased expression of Arg-1, IL-4R, IL-6, and YM-1; however, none were significant. (**B**) RAW264.7 cells co-cultured with DPSCs primed with LPS showed 1.5-fold increased gene expression of IL-1β, 1.5-fold increased expression of IL-4R, and 2-fold increased gene expression of YM-1. (**C**) RAW264.7 cells co-cultured with DPSCs primed with TNF-α showed 2.5-fold increased expression of IL-1β and 8-fold increased gene expression of IL-6. (**D**) RAW264.7 cells co-cultured with DPSCs primed with IFN-y showed 3-fold increased gene expression of IL-1B, 1.4-fold increased expression of TNF-a, 8-fold increased gene expression of IL-6, and 2.5-fold increased gene expression of IL-4R. Statistical significance was defined as (*) = *p* ˂ 0.05.

To determine the effect of primed DPSCs (with LPS, TNF-α, or IFN-γ for 4 h) on monocyte polarization, various gene expressions related to cell polarization in monocytes were determined using quantitative RT-PCR after 24 h of co-culture. For M1 polarization, the proinflammatory genes IL-1β and TNF-α were evaluated, and ARG-1, IL-4R, IL-6, and YM-1 were evaluated for M2 polarization. After 24 h of co-culture with DPSCs (without priming), the macrophages showed increased expression of all M2 genes, with significant increases in YM-1 expression (Figure 5A). Co-culture with DPSCs primed with LPS showed significantly increased expression of M2 genes IL-4R and YM-1 (Figure 5B). In addition, the co-culture of DPSCs primed with TNF-α showed significantly increased expression of the M2 gene IL-6 (Figure 5C). Finally, the co-culture of DPSCs primed with IFN-γ showed significantly increased expression of M2 genes IL-6 and IL-4R (Figure 5D). 

Among the M1 genes (IL-1β and TNF-α) tested, we found that the level of IL-1β was significantly increased in monocytes that were co-cultured with either primed or unprimed DPSCs (Figure 5A–D). The increased fold expressions were consistent among the groups (the fold increase being approximately 2-fold). However, the expression of TNF-α was only significantly increased in monocytes that were co-cultured with IFN-γ-primed DPSCs (Figure 5D).

### 3.7. Effect of Primed DPSCs on Monocyte Polarization-Related Protein Expression

**Figure 6:**
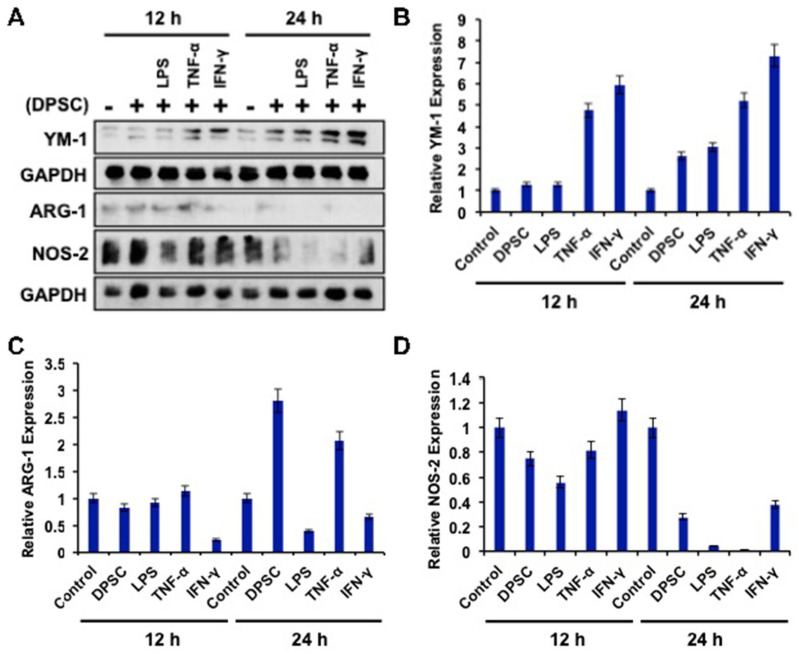
(**A**–**D**) RAW264.7 cells co-cultured with DPSCs and primed DPSCs showed increased expression of the protein YM-1 compared with the control at 12 and 24 h of co-culture. There was no significant change in Arg-1 in any of the groups at 12 or 24 h. Nos-2 protein expression was decreased in the co-culture groups at 24 h compared to the control.

To determine the effects of primed DPSCs (with LPS, TNF-α, or IFN-γ for 4 h) on monocyte polarization, YM-1, Arg-1 (for M2), and NOS2 (for M1) protein expressions, which are related to cell polarization in monocytes, were determined using WB after 12 and 24 h of co-culture. Monocytes co-cultured with DPSCs and primed DPSCs for 24 h showed less expression of Nos-2 and increased expression of YM-1 compared to the control (Figure 6A,B,D). Monocytes co-cultured with DPSCs and primed DPSCs for 12 h showed no remarkable decreases in the level of Nos-2 expression and remarkably increased expression of YM-1 in monocytes after priming DPSCs with TNF-α or IFN-γ when compared to the control (Figure 6A,B,D). In addition, Arg-1 was increased in the monocytes that were co-cultured with DPSCs or DPSCs primed with TNF-α compared to the control at 24 h (Figure 6A,C). However, monocytes co-cultured with DPSCs and primed DPSCs for 12 h showed no remarkable differences in the level of Arg-1 expression, except for monocytes that were co-cultured with IFN-γ primed DPSCs, in which the levels of Arg-1 were remarkably decreased (Figure 6A,C). These results indicated that monocytes co-cultured with DPSCs (and especially primed DPSCs) were induced toward M2 polarization.

### 3.8. Effect of Kynurenine on Monocytes

We observed that our DPSC primed with LPS had greater IDO expression compared to the control (Figure 3B,C). IDO is an enzyme involved in the breakdown of tryptophan. One of the byproducts of this breakdown is kynurenine [20]. Using a kynurenine assay, we measured the amount of kynurenine in DPSC media and found that, regardless of priming, the DPSCs secreted the same amount of kynurenine (Supplementary Figure S3). We hypothesized that this was due to the starting amount of tryptophan in the media. If unprimed DPSCs were already converting all the available tryptophan into kynurenine, then increasing the expression levels of IDO would not make a difference. We chose to continue to investigate this molecule as we did see M2 polarization in the RAW264.7 cells in both DPSCs and primed DPSCs. 

**Figure 7:**
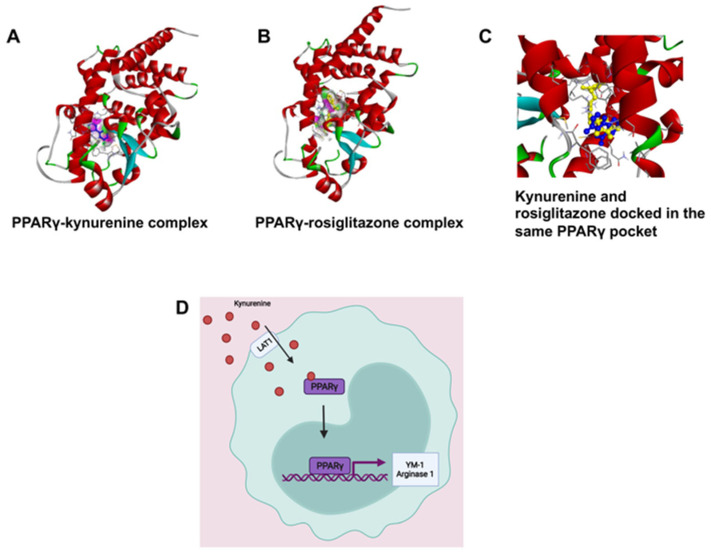
(**A**,**B**) Kynurenine-dependent transactivation of PPARγ, shown by molecular docking analysis and validated by comparing the interaction pattern of rosiglitazone with PPARγ. (**C**) The molecular overlay of the kynurenine-PPARγ complex and rosiglitazone-PPARγ complex indicates that both bind in the LBD of PPARγ. (**D**) We hypothesize that kynurenine is being transported into the RAW264.7 cell by LAT1 and binding to PPARγ causing its activation to move into the nucleus and induce transcription of the genes YM-1 and Arg-1.

PPARγ is a transcription factor that induces M2 polarization [21]. Kynurenine-dependent transactivation of PPARγ was predicted by molecular docking analysis; the results were validated by comparing the interaction pattern of rosiglitazone with PPARγ. Kynurenine was firmly docked into the ligand-binding domain (LBD) with significant binding energy of −6.15 kcal/mol, which was close to the known PPARγ agonist, rosiglitazone (−7.87 kcal/mol). Interestingly, the molecular overlay of the kynurenine-PPARγ complex and the rosiglitazone-PPARγ complex indicated that both bound in the LBD of PPARγ (Figure 7 and Figure S4). The kynurenine-PPARγ complex was stabilized with six hydrogen bonds, while rosiglitazone formed four hydrogen bonds with PPARγ (Supplementary Figure S5). The detailed analysis of binding amino acid residues as well as their nature of interactions are summarized in a supplementary table (Supplementary Figure S5). A higher number of hydrogen bonds in the kynurenine-PPARγ, complex compared to the rosiglitazone-PPARγ complex, revealed markedly stable interaction. The obtained results indicated that kynurenine may act as an agonist of PPARγ. 

**Figure 8:**
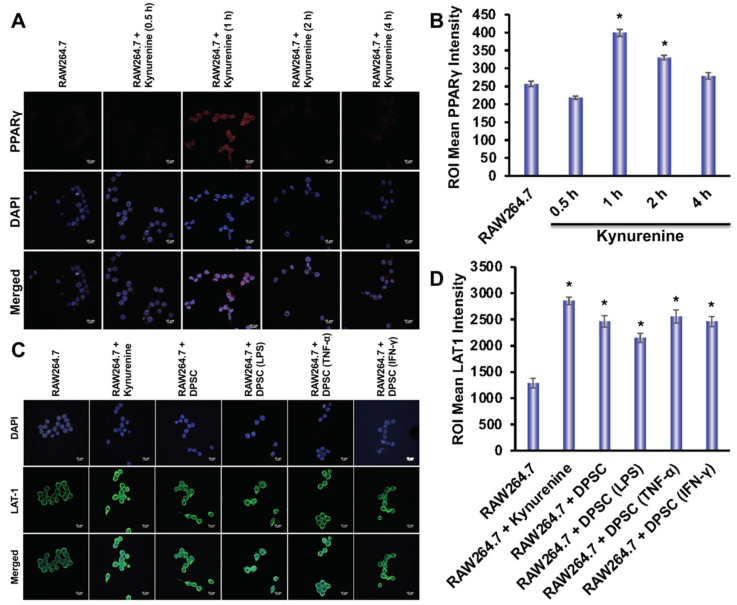
(**A**,**B**) Kynurenine stimulation of RAW264.7 cells increases protein expression of PPARγ at the 1 h time point with decreasing expression at the 2 h and 4 h time point. (**C**,**D**) RAW264.7 cells stimulated with kynurenine for 24 h or co-cultured with DPSCs or primed DPSCs for 24 h showed increased expression of the LAT1 transporter. Statistical significance was defined as (*) = *p* ˂ 0.05.

Using immunofluorescence, we evaluated the expression of LAT-1 and PPARγ in RAW264.7 cells. The expression of LAT-1 is critical for the transportation of kynurenine into the cells [22]. We designed six groups to determine the expression of LAT-1 in RAW264.7 cells in the presence or absence of kynurenine or presence or absence of co-culture with DPSCs, either primed or unprimed. Those groups were: control, RAW264.7 and kynurenine, RAW264.7 co-cultured with DPSC, RAW264.7 co-cultured with DPSC primed with LPS, RAW264.7 co-cultured with DPSC primed with TNF-α, and RAW264.7 co-cultured with DPSC primed with IFN-γ. RAW264.7 cells were co-cultured with DPSCs in a trans-well insert or exposed to 20 μM of kynurenine for 24 h, which was optimized in our culture system. We observed an increased expression of LAT-1 in all groups compared to control, but especially the RAW264.7 and kynurenine group and the RAW264.7 co-cultured with DPSC primed with TNF-α group (Figure 8B). We also tested PPARγ expression under these conditions, but did not observe any increase (data not shown). Therefore, tested RAW264.7 expression of PPARγ under kynurenine (20 µM) stimulation at earlier various time points. We checked PPARγ expression after 30 min, 1 h, 2 h, and 4 h of kynurenine stimulation and found a significant increase in expression at the 1 h time point with decreasing levels at the 2 h and 4 h timepoints (Figure 8A). 

## 4. Discussion

The goal of this project was to determine if primed DPSCs were more efficacious than unprimed DPSCs for the future treatment of inflammation, and if so, what mechanisms underlaid the effects. The multiplex ELISA data showed that the DPSCs had an inherent anti-inflammatory profile. However, we wanted to see if that profile could be enhanced through priming. After 4 h of priming with LPS, TNF-α, or IFN-γ, the DPSCs showed an increased genetic expression of IDO, HGF, IL-4, and IL-10. Since the level of expression and significance varied depending on which stimulant was used, we chose to go forward with all of them. 

We also needed to see if priming affected the health of the DPSCs. Flow cytometry showed no difference in stem cell markers between control and priming. However, the MitoStress assay showed that priming the DPSCs, especially with TNF-α or IFN-γ, decreased the metabolic activity, as represented by decreased ATP production and decreased basal respiration. The ability of the DPSCs to respond to metabolic demand was also decreased, as shown by lower spare respiratory capacity and maximal respiration. Therefore, priming DPSCs with TNF-α or IFN-γ decreased the amount of energy produced by the mitochondria as well as the mitochondria’s ability to produce more energy if needed.

Our next step was to evaluate the effects of DPSCs on wound healing. We used gut epithelial cells co-cultured with DPSCs, either primed or without priming. These results showed a significantly greater wound closure with DPSCs, either primed or unprimed, compared to control where no DPSCs were used. This was likely due to the ability of DPSCs (and MSCs, more broadly) to secrete growth factors such as basic fibroblast growth factor, transforming growth factor-β, platelet derived growth factor and TSG-6, all of which can promote cell proliferation, thereby mediating wound healing [23,24]. The WB showed a decrease in levels of pAkt, pp65, and increased pERK1/2 expression after co-culture with DPSCs. These results showed that DPSCs were secreting factors that inhibited the Akt pathway, in which p65 is a downstream target, while enhancing the ERK 1/2 pathway. Akt and ERK 1/2 are well known for being involved in cell proliferation, migration, and growth [25]. We hypothesized that the greater wound closure observed in our DPSC co-culture groups was due to the secretion of factors that upregulated the ERK 1/2 pathway. This was supported by the literature, which showed that the ERK 1/2 was upregulated in LoVo cells as a result of galectin-3 secretion by MSCs, which increased cell proliferation [26]. However, it was interesting that the Akt–p65 pathway was downregulated, as it demonstrated secretion of anti-inflammatory factors, in line with the other data we presented. This result also supported previous data showing that CD34+ stem cells also accelerated wound closure via upregulation of angiogenic factors [27].

In the next set of experiments, we looked at the effects of the DPSCs and primed DPSCs on monocyte polarization. Monocytes have a pro-inflammatory M1 and an anti-inflammatory M2 profile. We observed that the monocytes co-cultured with unprimed DPSCs trended toward M2-related gene expressions—more precisely, with a significantly increased YM-1 expression. Monocytes co-cultured with DPSCs primed with LPS, TNF-α, or IFN-γ also had significantly increased gene expression in the M2 genes. WB analysis confirmed the increased expression of YM-1 in monocytes co-cultured with primed DPSCs. Therefore, DPSCs and primed DPSCs were efficacious in inducing M2 polarization in monocytes. These data suggested that the primed DPSCs were slightly more effective in inducing M2 polarization. We concluded that DPSCs could be secreting factors, such as proteins and microRNAs, inducing monocytes toward M2 polarization, as demonstrated by the increased expression of the genes and proteins IL-4R, Arg1, YM-1 and IL-6.

IDO is an important enzyme that is responsible for the breakdown of tryptophan. We wanted to explore the role of IDO in immune polarization, as both gene and protein expressions were significantly increased with the priming of DPSCs. One of the metabolites produced from this pathway was kynurenine. Kynurenine is a ligand for the aryl hydrocarbon receptor, a transcription factor that has been shown to play a critical role in the polarization of macrophages into the M1/M2 phenotypes [28]. We found, through in silico modeling, that kynurenine wsa a potential agonist for PPARγ, a transcription factor that can induce M2 polarization in monocytes through induction of genes including CD206, Arg-1, YM-1, FIZZ-1 as well as inducing retinoic acid signaling (which is also necessary for M2 polarization) [21,29]. We found that RAW264.7 cells stimulated with kynurenine, co-cultured with DPSCs, or primed DPSCs, had greater expression of the LAT-1 protein, the transporter for kynurenine, which helps to transport kynurenine into the cells. We also found that kynurenine increased the expression of the transcription factor PPARγ after one hour of stimulation with kynurenine in the monocytes. 

Given that we knew DPSCs secreted kynurenine, that kynurenine was being transported into the cell, and that PPARγ expression increased with kynurenine stimulation, we hypothesized two mechanisms by which PPARγ was being induced. The first was the most straightforward: that kynurenine was transported into the cell and acted as an inducer to PPARγ, increasing its expression. The second was more circuitous. Our in silico modeling also showed that kynurenine was a potential antagonist of PTP1b, a phosphatase that regulates M1/M2 polarization. One of its targets was STAT6 (data not shown). Phosphorylated STAT6 can both act as a transcription factor for M2 polarization and induce PPARγ [30,31]. Discovering which pathway caused the results we observed was, unfortunately, outside of the scope of this paper, but is something we would like to study in the future.

The goal of this paper was to investigate DPSCs and primed DPSCs as a therapeutic option for the treatment of inflammation. Our results indicated that, while priming DPSCs with LPS, TNF-α, or IFN-γ could induce healing, it was not necessarily a better option than unprimed DPSCs. RAW264.7 cells co-cultured with primed DPSCs showed slightly greater M2 polarization compared to RAW264.7 cells co-cultured with DPSCs. However, there was no difference in wound closure rates when comparing the DPSC group to the primed DPSC groups. Additionally, primed DPSCs showed decreased mitochondrial function. However, one of the novel discoveries of this research was a mechanism by which DPSCs and primed DPSCs were inducing M2 polarization through the IDO-kynurenine pathway. We found that kynurenine was a potential inducer of PPARγ, and increased expression of this transcription factor was likely one of the pathways by which DPSCs promoted M2 polarization in RAW264.7 cells. In the future, we would like to apply this research to a disease model of inflammatory bowel disease. MSCs have the potential to induce M2 polarization in macrophages in murine colitis models, and have exerted this effect to facilitate wound healing [21]. Our data support the theory that DPSCs would exert the same effect. However, DPSCs are unique, in that they have greater potential to heal the neuronal damage that contributes to chronic pain in patients with IBD [32,33].

## Data Availability

Not applicable.

## Supplementary Materials

The following supporting information can be downloaded at: https://www.mdpi.com/article/10.3390/biomedicines10081999/s1, Figure S1: Stem cell markers; Figure S2: Representative graph of Seahorse analysis; Figure S3: Kynurenine Assay results; Figure S4: In silico modeling of PPARγ-kynurenine complex and PPARγ-rosiglitazone complex; Figure S5: Docking analysis of kynurenine and rosiglitazone with PPARγ; Table S1: List of mouse primer used in this study; Table S2: List of human primer used in this study.
