# Supplementary material for: Dental Pulp-Derived Stem Cells Reduce Inflammation, Accelerate Wound Healing and Mediate M2 Polarization of Myeloid Cells
Source: Biomedicines. 2022 Aug 17;10(8):1999. doi: 10.3390/biomedicines10081999 (PMC9624276; doi:10.3390/biomedicines10081999)
Supplement: Supplementary file 1 [file biomedicines-10-01999-s001.zip › biomedicines-1761755-SI.pdf]

biomedicines

MDPI

Supplementary Figures

Dental Pulp-Derived Stem Cells Reduce Inflammation, Accelerate Wound Healing, and Mediate M2 Polarization of Myeloid Cells

Sarah Anderson, Prateeksha Prateeksha and Hiranmoy Das *

Department of Pharmaceutical Sciences, Jerry H. Hodge School of Pharmacy, Texas Tech University Health Sciences Center, Amarillo, TX 79106, USA
* Correspondence: hiranmoy.das@ttuhsc.edu

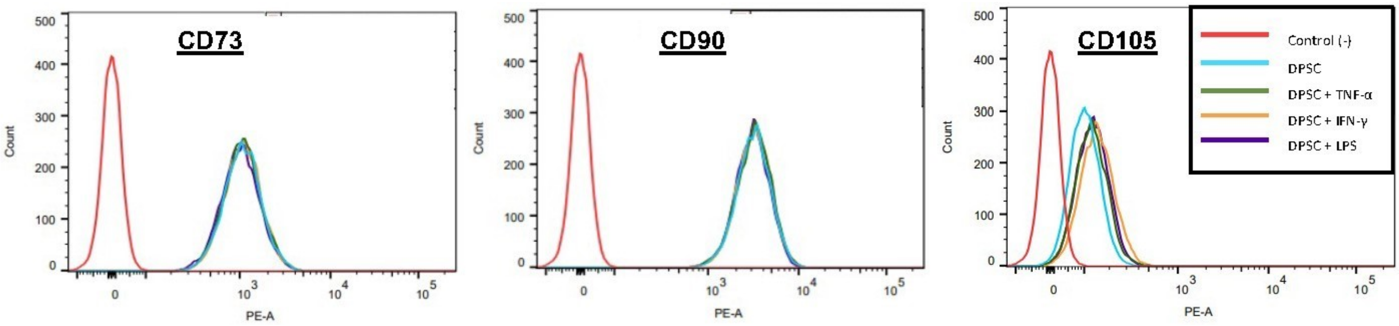

Figure S1: Stem cell markers.

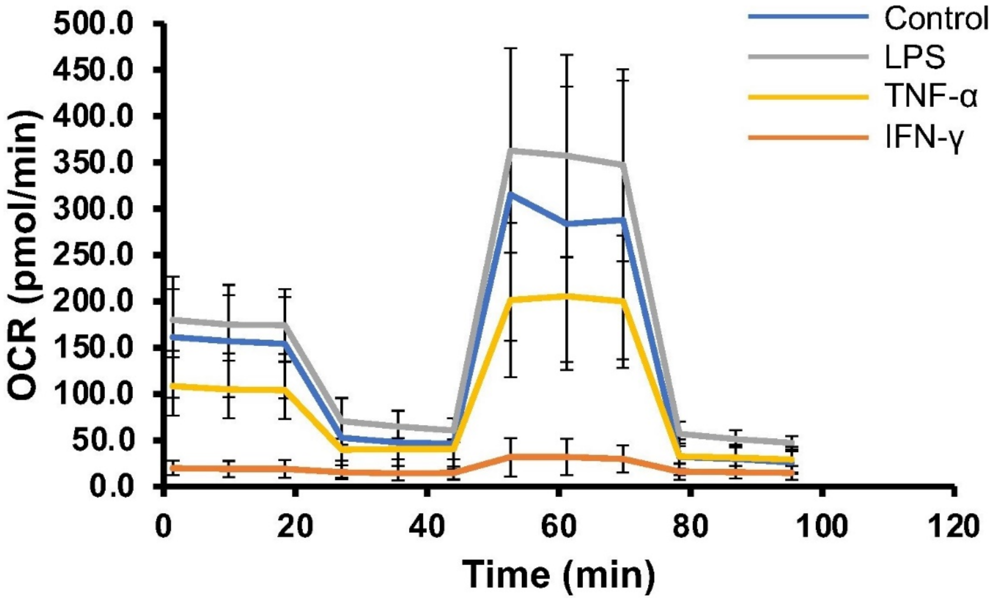

Figure S2: Representative graph of Seahorse analysis.

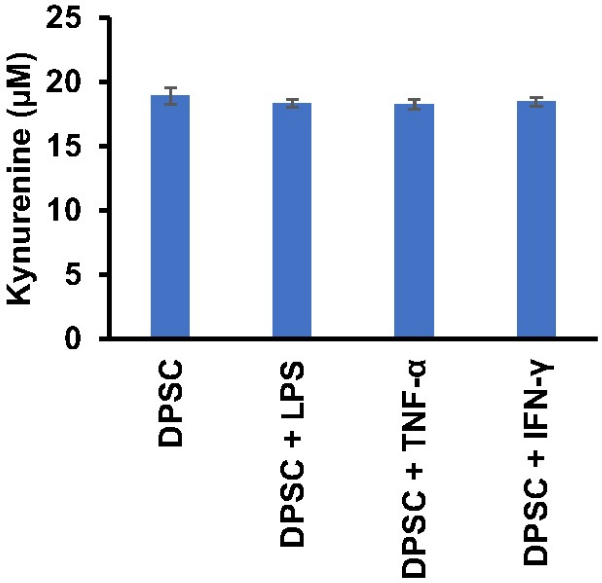

Figure S3: Kynurenine Assay results.

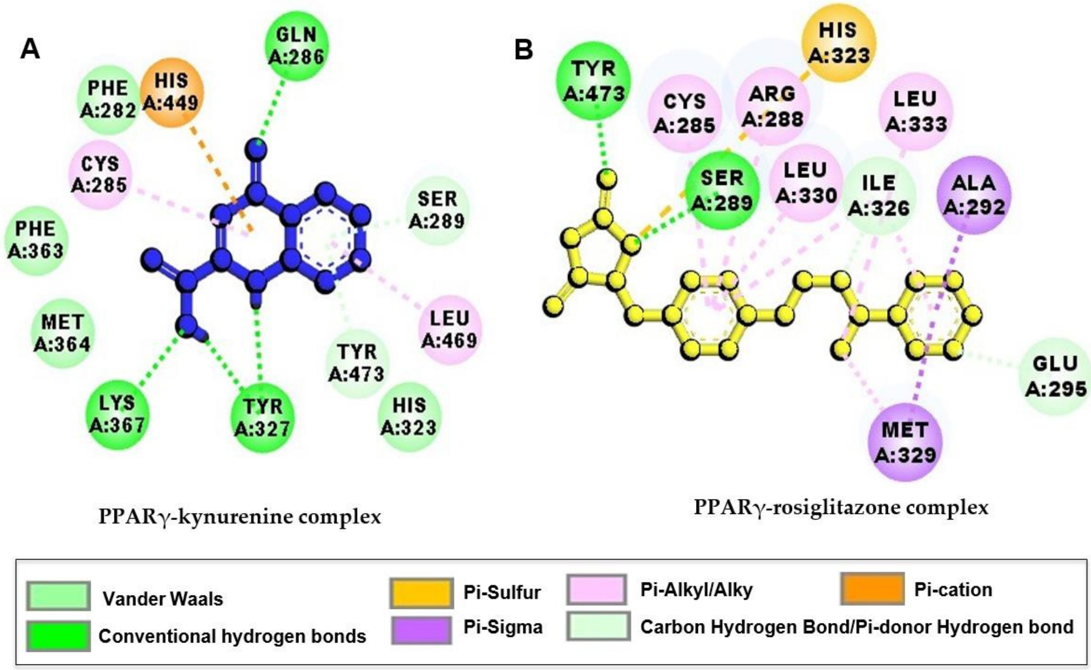

Figure S4: In silico modeling of PPARγ-kynurenine complex and PPARγ-rosiglitazone complex.

Molecules	Binding Affinity (kcal/Mol)	Type of Interactions	Interacting Residues	Distance (Å)
**Kynurenine**	-6.15	H-bond	Lys367	2.23
		H-bond	Tyr327	2.07, 1.92
		H-bond	Gln286	3.06
		**Pi-Donor H-bond**	**Ser289**	**2.77**
		**Pi-Donor H-bond**	**Tyr473**	**2.80**
		**Pi-Alkyl**	**Cys285**	**4.59**
		Pi-Alkyl	Leu469	5.13
		Pi-Cation	His449	4.16
**Rosiglitazone (Agonist)**	-7.87	**H-bond**	**Ser 289**	**2.27**
		**H-bond**	**Tyr473**	**2.05**
		Carbon Hydrogen Bond	Ile326	3.00
		Carbon Hydrogen Bond	Glu295	3.00
		**Alkyl/Pi-alkyl**	**Cys285**	**4.80**
		Alkyl/Pi-alkyl	Arg288	4.99
		Alkyl/Pi-alkyl	Leu330	4.92
		Alkyl/Pi-alkyl	Leu333	4.64
		Alkyl/Pi-Alkyl	Ile326	5.07
		Pi-Sulfur	His323	5.79
		Pi-Sigma	Ala292	3.40
		Pi-Sigma	Met329	3.61

Figure S5: Docking analysis of kynurenine and rosiglitazone with PPARγ.

Table S1: List of mouse primer used in this study.

Mouse primers		
	**Forward**	**Reverse**
**IL-1β**	5′ TGG AGA GTG ATC CCA AGC AAT 3′	5′ TGC TTG TGA GGT GCT GAT GTA CCA 3′
**TNF-α**	5′ TTC CGA ATT CAC TGG AGC CTC GAA 3′	5′ TGC ACC TCA GGG AAG AAT CTG GAA 3′
**Arg-1**	5′ CCA GGG ACT GAC TAC CTT AAA C 3′	5′ GAA GGC GTT TGC TTA GTT CTG 3′
**YM-1**	5′ GCT AAG GAC AGG CCA ATA GAA 3′	5′ GCA TTC CAG CAA AGG CAT AG 3′
**IL-6**	5′ ATC CAG TTG CCT TCT TGG GAC TGA 3′	5′ TAA GCC TCC GAC TTG TGA AGT GGT 3′
**IL-4R**	5′ CAA GCT CTG ACC TCT GGA TTA G 3′	5′ AAT GAT GGG AGC GGG TAT AAG 3′
**β-actin**	5′ AAT GTG GCT GAG GAC TTT GT 3′	5′ GGG ACAT TCC TGT AAC CAC TTA TT 3′

Table S2: List of human primer used in this study.

Human Primers		
	**Forward**	**Reverse**
**HGF**	5′ ATC AAA TGT CAG CCC TGG AG 3′	5′ TCG ATA ACT CTC CCC ATT GC 3′
**IL-4R**	5′ GTT CTA CAG CCA CCA TGA GAA 3′	5′ CCG TTT CAG GAA TCG GAT CA 3′
**IDO**	5′ AGG ATT CTT CCT GGT CTC TCT 3′	5′ GTG TCC CGT TCT TGC ATT TG 3′
**IL-10**	5′ TGA GCT TCT CTG TGA ACG ATT TA 3′	5′ GTC ACC CTA TGG AAA CAG CTT A 3′
**GAPDH**	5′ ATG ACA AGC TTC CCG TTC TC 3′	5′ CCC TTC ATT GAC CTC AAC TAC A 3′
